# Supplementary material for: Crystal structure of Zen4 in the apo state reveals a missing conformation of kinesin
Source: Nat Commun. 2017 Apr 10;8:14951. doi: 10.1038/ncomms14951 (PMC5394238; doi:10.1038/ncomms14951)
Supplement: Supplementary Information — Supplementary Figures and Supplementary References [file ncomms14951-s1.pdf]

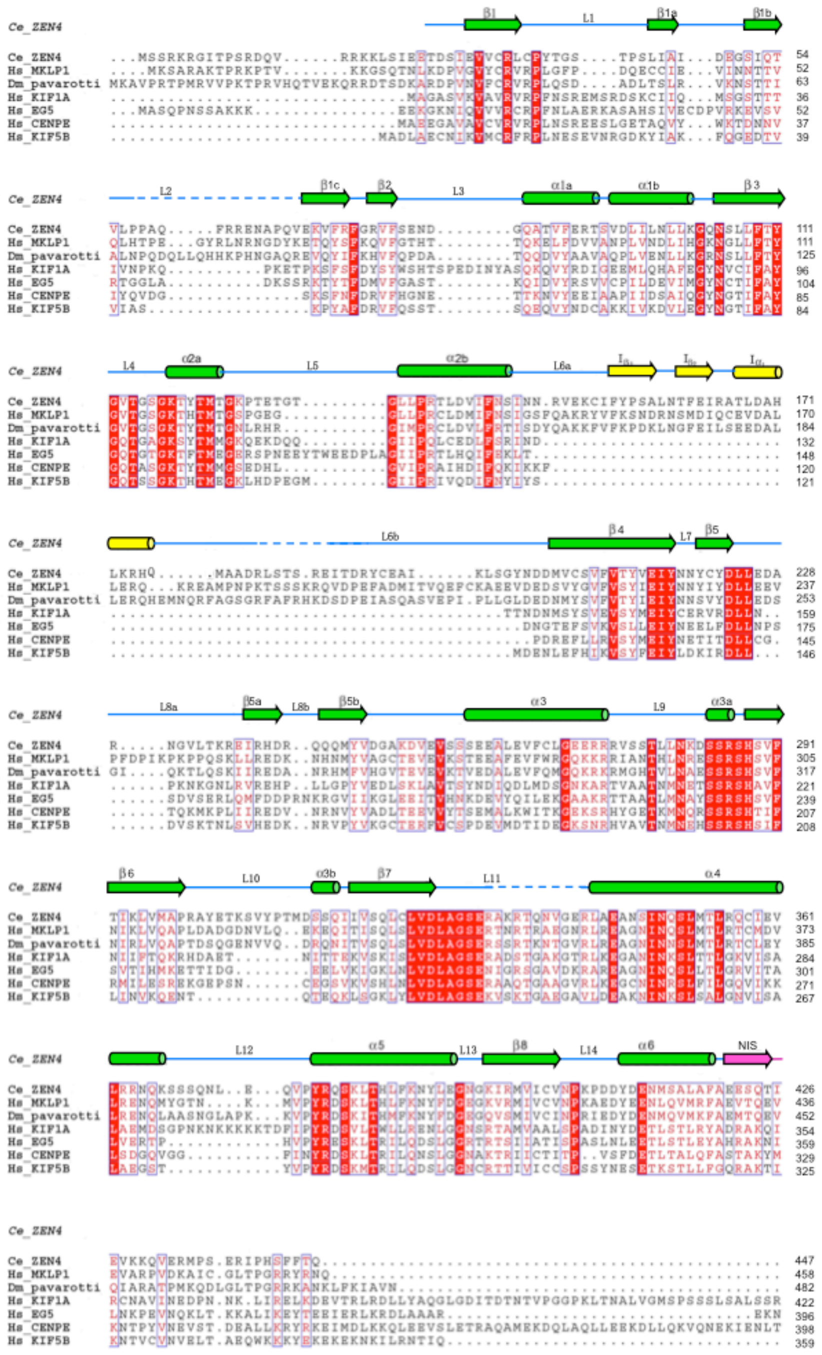

Supplementary Fig. 1 Sequence alignments of three kinesin-6 family proteins with human KIF1A (kinesin-3), EG5 (Kinesin-5), CENPE (kinesin-10) and KIF5B (Kinesin-1). The secondary structure assignments are based on the structure determined here. Identical residues are highlighted with red bars, and similar residues are colored red.

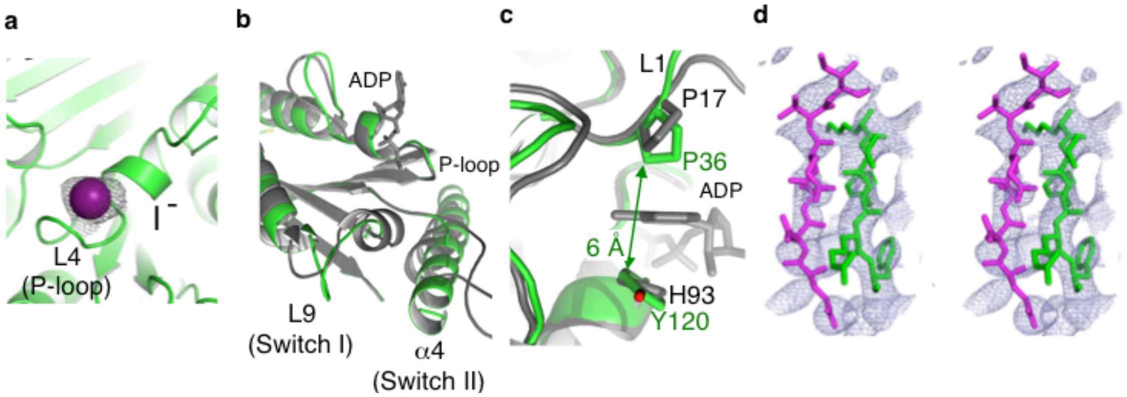

Supplementary Fig. 2 Structural features of Zen4 in the apo state

(**a**) Superimposition of the structure of Zen4 around the P-loop and the Fo-Fc difference map at a contour level of σ=2 with the bound ion removed. The bound ion is most likely to be iodine (I^-), which is present in the crystallization solution. (**b-c**) Superimposition of the structure of Zen4 and that of kinesin-1 in the ADP state (grey, PDB code 1BG2), showing regions around the switch region (**b**) and the ATP-binding pocket (**c**). The alignments were done on the N-terminal P-loop subdomain. (**d**) Stereo images of the structure of Zen1 around the NIS (magenta) superimposed with the 2Fo-Fc map (blue) at σ=2.

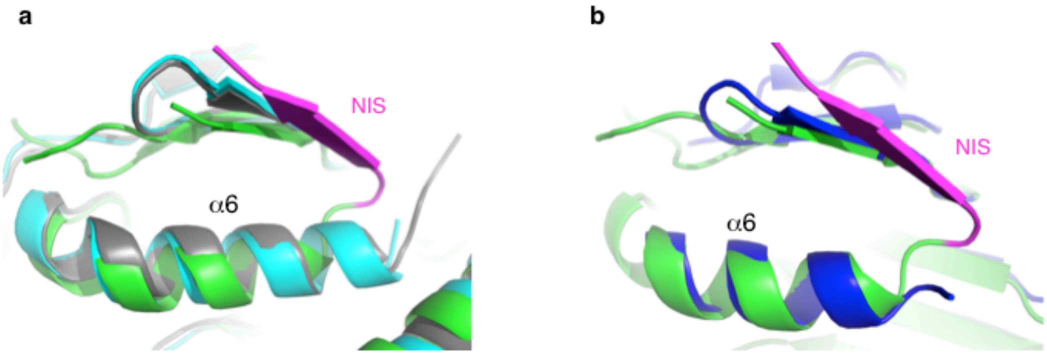

Supplementary Fig. 3 Comparisons of the structure of α6 at different states (**a**) Superimpositions of the crystal structure of Zen4 (green) with those of kinesin-1 in the ADP-bound state (grey, PDB code 1BG2) and apo state (cyan, PDB code 4LNU) around the α6 helix. Comparing with the reported crystal structures of kinesin-1 (PDB codes 1BG2 and 4LNU), the α6 helix of Zen4 is almost one helical turn shorter at the C-terminal end. (**b**) Superimposition of the crystal structure of Zen4 (green) with the cryoEM structure of kinesin-1 in the apo state (blue, PDB code 3J8X) around the α6 helix. The C-terminal ends of α6 helixes are partially melted in both structures.

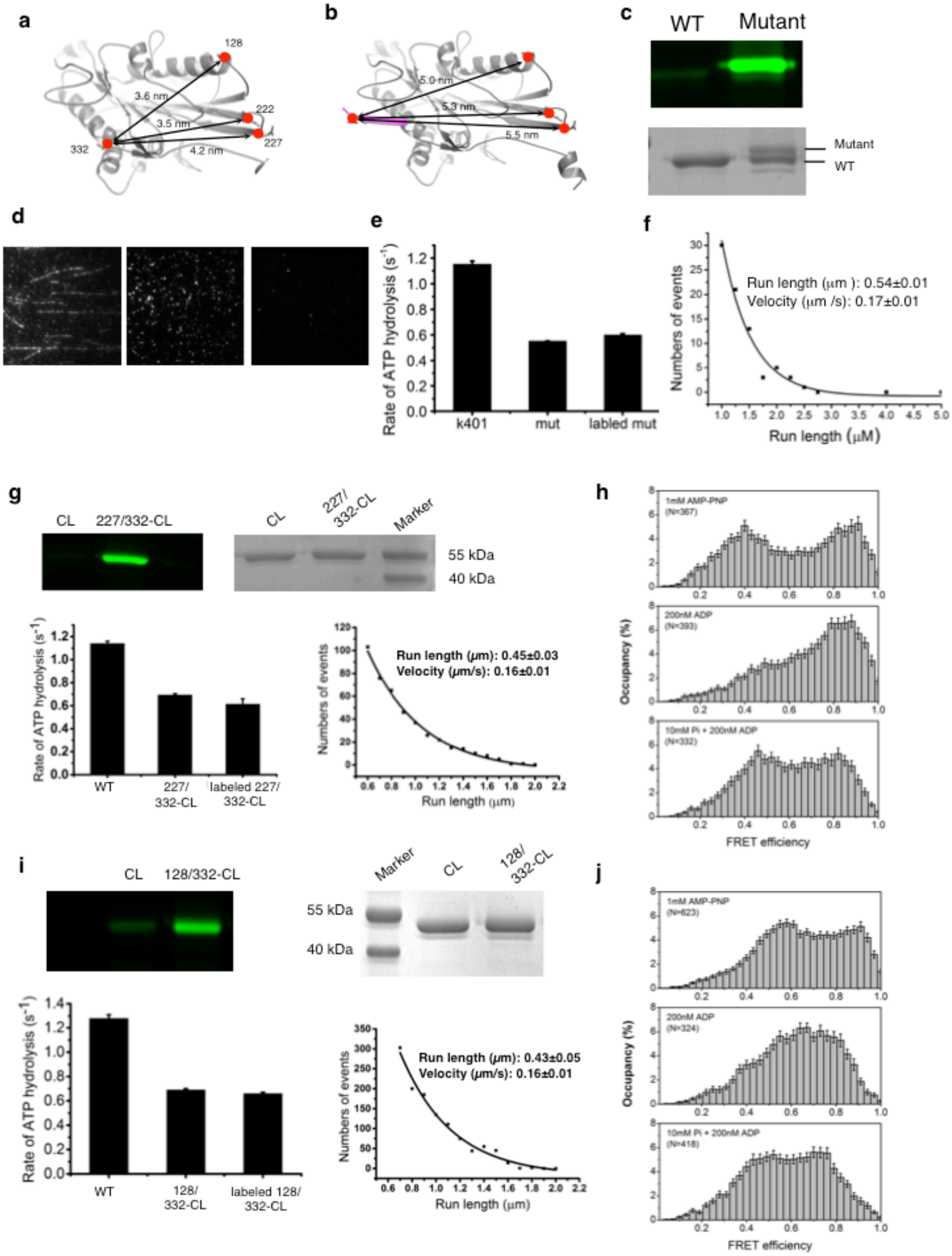

Supplementary Fig. 4 Specificities in dye labeling, surface immobilization and activities of *Drosophila* kinesin-1 (1-401).

(**a**) Structure of human kinesin-1 in ATP-bound state (PDB code 3J8Y). The positions of the residues for dye labeling are shown as red dots. The numbers show the equivalent residues of *Drosophila* kinesin-1. The estimated distances of the three FRET sensor pairs: 128/332: 3.6 nm; 222/332, 3.5 nm; 227/332, 4.2 nm. The estimated distances of the three FRET sensor pairs in the ADP would be similar as those in the ATP state. (**b**) Proposed structure of kinesin-1 in apo state with a backward docked NIS (in magenta). The estimated distances of the three FRET sensor pairs: 128/332: 5.0 nm; 222/332, 5.3 nm; 227/332, 5.5 nm. (**c**) Site-specific dye-labeling of kinesin with unnatural amino residue. DmKhc(1-401) and the mutant (E222TAG/T332TAG) were labeled with DBCO-sulfo-Cy3 for 1h at room temperature, followed by running 15% gel. The gel was imaged upon excitation of the fluorophore Cy3 immediately after running. The bottom panel shows the same gel stained with Coomassie brilliant blue. The mutant contains a C-terminal Flag-tag, and migrated more slowly than the WT protein in the SDS-PAGE gel. (**d**) Specificity of surface immobilization on coverslips. The surfaces of coverslips with biotin-PEG were incubated with streptavidin and biotin-microtubules (left and middle panels) or streptavidin alone (right panel). Dye-labeled kinesins were added and incubated for 1 min, and then washed twice. When a high concentration of kinesin (>20 nM) was used, the molecules were found to bind MT and form a line (left panel). To collect the FRET signals and resolve individual molecules, a lower concentration of kinesin (~10 nM) was used (middle panel). Very few molecules on the surface were found if microtubules were absence (right panel). (**e**) Microtubule-stimulating ATPase activity of WT, and the mutant carrying the unnatural amino acid residues before (Mut) and after (Labeled-mut) dye-labeling. The mutant retained ~50% of the ATPase activity of the WT protein, and the dyes did not perturb the activity. (**f**) The labeled mutant is active in the single molecular mobility assays, with the characteristic run length of ~0.54 μm and velocity of ~0.17 μm/s. The magnitudes of run length and velocity of the labeled mutant are ~50% of the reported values reported for the WT protein[1-3]. (**g**) Activity of the cysteine-light (CL) drosophila kinesin-1 mutant with the FRET sensor pair at 227/332. Top left panel, site-specificity of dye-labeling; top right panel, the same gel stained with Coomassie brilliant blue; bottom left panel, microtubule-stimulating ATPase activity of WT and the mutant; bottom right panel, single molecular mobility assays. The labeled mutant showed a characteristic run length of ~0.45 μm and velocity of ~0.16 μm/s. (**h**) Histograms of FRET efficiency of the CL kinesin mutant with the FRET sensor pair at 227/332 bound to microtubule in 1 mM AMP-PNP (top panel), 200 nM ADP (middle panel) and 10 mM Pi + 200 nM ADP (bottom panel), respectively. (**i**) Activity of the cysteine-light (CL) drosophila kinesin-1 mutant with the FRET pair at 128/332. The experiments were performed as in (**g**). The labeled mutant showed a characteristic run length of ~0.43 μm and velocity of ~0.16 μm/s. (**j**) Histograms of FRET efficiency of the CL kinesin mutant with the FRET pair at 128/332. The experiments were performed as in (**h**).

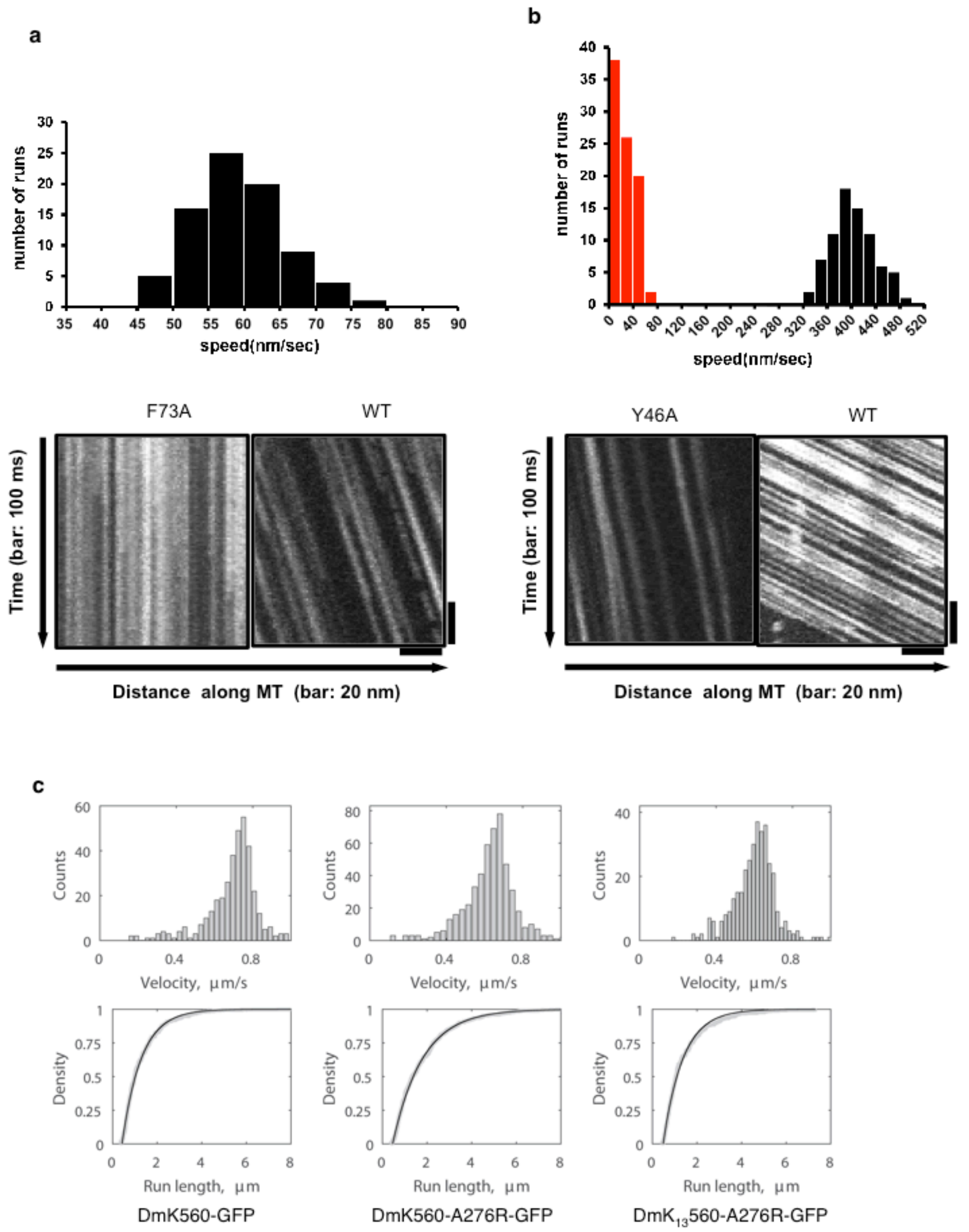

Supplementary Fig. 5 Pairing with the N-terminal appending β-sheet is important for the motility of both Zen4 and Kinesin-1. (**a**) Microtubule gliding driven by Zen4. Top panel, distribution of the gliding speed driven by WT Zen4. Disruption of the pairing with the N-terminal appending β-sheet through mutation F73A completely disrupted microtubule gliding. The bottom panels show the kymographs of the WT Z601 and the F73A mutant. **(b**) Microtubule gliding driven by rat kinesin-1. Top panel, distribution of the gliding speed driven by WT kinesin-1 (black bars) and the Y46A mutant (red bars).

The bottom panels show the kymographs of the WT and the Y46A mutant. (**c**) Enhanced processicivity of drosophila kinesin-1 (1-560) due to introduction of the "arginine gate" mutation (A276R). DmK560-GFP, WT drosophila kinesin-1 (1-560) tagged with GFP; DmK560-A276R-GFP, mutant kinesin-1 with the "arginine gate" mutation (A276R); DmK_{13}560-A276R-GFP, shortened NL kinesin-1 mutant[4] with the "arginine gate" mutation (A276R). The run lengths of these three proteins are 0.86±0.12 (N=365), 1.3±0.14 (N=511) and 0.89±0.11 μm (N=347), respectively (distributions in gray, fits in black). The velocities of these three proteins are 0.70±0.07, 0.63±0.06, and 0.60±0.06 $\mu m/s^{-1}$, respectively.

Reference:

1. Martin, D.S., Fathi, R., Mitchison, T.J. & Gelles, J. FRET measurements of kinesin neck orientation reveal a structural basis for processivity and asymmetry. *Proc. Natl. Acad. Sci. U S A* **107**, 5453-8 (2010).
2. Fehr, A.N., Gutierrez-Medina, B., Asbury, C.L. & Block, S.M. On the origin of kinesin limping. *Biophys J.* **97**, 1663-70 (2009).
3. Andreasson, J.O. et al. Examining kinesin processivity within a general gating framework. *Elife* **4**(2015).
4. Shastry, S. & Hancock, W.O. Neck linker length determines the degree of processivity in kinesin-1 and kinesin-2 motors. *Curr. Biol.* **20**, 939-43 (2010).
